# Intra- and interobserver reliability analysis of pediatric lower limb parameters on digital long leg radiographs

**DOI:** 10.1186/s13018-023-03552-8

**Published:** 2023-01-27

**Authors:** Sebastian Braun, Marco Brenneis, Jana Holder, Andrea Meurer, Felix Stief

**Affiliations:** 1 Department of Orthopedics (Friedrichsheim), University Hospital Frankfurt, Goethe University, 60528 Frankfurt/Main, Germany; 2 Dr. Rolf M. Schwiete Research Unit for Osteoarthritis, Department of Orthopedics (Friedrichsheim), University Hospital Frankfurt, Goethe University, Frankfurt/Main, Germany; 3 Medical Park St. Hubertus Klinik, Bad Wiessee, Germany; 4 Department of Sports and Exercise Science, University of Salzburg, 5020 Salzburg, Austria

**Keywords:** Lower limb deformities, Leg axis, Genu valgum, Radiological assessment, Reliability, Pediatric orthopedic, Guiding growth

## Abstract

**Background:**

Malalignments of the lower extremity are common reasons for orthopedic consultation because it may lead to osteoarthritis in adulthood. An accurate and reliable radiological assessment of lower limb alignment in children and adolescents is essential for clinical decision-making on treatment of limb deformities and for regular control after a surgical intervention.

**Objective:**

First, does the analysis of full-length standing anteroposterior radiographs show a good intra- and interobserver reliability? Second, which parameter is most susceptible to observer-dependent errors? Third, what is the Standard Error of Measurement (SEM_95%_) of the absolute femoral and tibial length?

**Methods:**

Two observers evaluated digital radiographs of 144 legs from 36 children and adolescents with pathological valgus alignment before a temporary hemiepiphysiodesis and before implant removal. Parameters included Mechanical Femorotibial Angle (MFA), Mechanical Axis Deviation (MAD), mechanical Lateral Distal Femoral Angle (mLDFA), mechanical Medial Proximal Tibial Angle (mMPTA), mechanical Lateral Proximal Femoral Angle (mLPFA), mechanical Lateral Distal Tibial Angle (mLDTA), Joint Line Convergence Angle (JLCA), femur length, tibial length. Intra- and interobserver reliability (ICC_2,1_), SEM_95%_ and proportional errors were calculated.

**Results:**

The intra- and interobserver reliability for almost all measurements was found to be good to excellent (Intra-ICC_2,1_: 0.849–0.999; Inter-ICC_2,1_: 0.864–0.996). The SEM_95%_ of both observers was found to be ± 1.39° (MFA), ± 3.31 mm (MAD), ± 1.06° (mLDFA) and ± 1.29° (mMPTA). The proportional error of MAD and MFA is comparable (47.29% vs. 46.33%). The relevant knee joint surface angles show a lower proportional error for mLDFA (42.40%) than for mMPTA (51.60%). JLCA has a proportional error of 138%. Furthermore, the SEM_95%_ for the absolute values of the femoral and tibial length was 4.53 mm for the femur and 3.12 mm for the tibia.

**Conclusions:**

In conclusion, a precise malalignment measurement and the knowledge about SEM_95%_ of the respective parameters are crucial for correct surgical or nonsurgical treatment. The susceptibility to error must be considered when interpreting malalignment analysis and must be considered when planning a surgical intervention. The results of the present study elucidate that MAD and MFA are equally susceptible to observer-dependent errors. This study shows good to excellent intra- and interobserver ICCs for all leg alignment parameters and joint surface angles, except for JLCA.

*Trial registration*: This study was registered with DRKS (German Clinical Trials Register) under the number DRKS00015053.

**Level of evidence:**

I, Diagnostic Study.

## Background

Osteoarthritis (OA) of the knee is a common cause for knee pain, reduced joint motion and consecutive muscle weakness leading to physical inactivity, reduction in quality of life, disability and eventually a total joint replacement [1]. Varus or valgus malalignment of the lower extremity has a major impact on the development and progression of knee OA [2]. The valgus deformity leads to increased load on the lateral compartment of the knee joint and the varus malalignment increases the load on the medial compartment [3–6].

To prevent the development of OA, growth control should be considered in children with severe axial deviation in the frontal plane. For children and adolescents, there is a wide range of normal values concerning axis deviation of the lower limb, which need to be differentiated from pathological and pre-arthritic deformities prior to surgical intervention [7]. As a surgical treatment, temporary hemiepiphysiodesis (THE) of the distal femur or proximal tibia using tension band plates, depending on the location of the pathologic joint surface angles, has become established for axis correction of children and adolescents [8, 9]. However, the exact patient age for the best possible correction and the lowest probability of rebound remains unclear. Furthermore, there is a lack of defined parameters for the indication of THE. The definition of parameters like the Mechanical Axis Deviation (MAD) or the Mechanical Femorotibial Angle (MFA) is complicated by the interindividual variation during growth, the uncertainty of when MAD leads to pathological joint moments and pressure loads as well as the determination method and the associated susceptibility to errors [4, 5, 10, 11].

Malalignment of the lower extremity is one of the most common reasons for an orthopedic consultation. An accurate and reliable radiological assessment of lower limb alignment is essential for clinical decision-making on treatment of limb deformities and for regular control after a surgical intervention [8, 12]. The full-length standing anteroposterior radiograph of both legs is the gold standard for assessment of lower limb alignment in frontal plane [13]. Most commonly the indication for a THE is set when the physiological mechanical bearing line (MAD) deviates more than 10 mm lateral or 15 mm medial to the center of the knee [14], which is approximately 3° deviation of the physiological MFA. Furthermore, knowledge of the joint surface angles and the Joint Line Convergence Angle (JLCA) is important to assess on which bones the guided growth intervention should be performed. In addition, an estimate of the residual growth can be made based on the absolute lengths of the femur or tibia in relation to age and sex and thus the correction potential of the guided growth intervention can be determined [15–17].

To the best of our knowledge no study has shown which parameter or combination of parameters is more reliable for setting the indication for a THE in children and adolescents. Therefore, we first aim to elucidate if the analysis of a full-length standing anteroposterior radiograph of both legs shows a good intra- and interobserver reliability concerning lower limb alignment measurements in the frontal plane. A misjudgment or incorrect determination may result in a delayed or unnecessary indication for THE and thus in an incorrect manipulation of the leg axis. Second, it is important to clarify which of the investigated alignment parameters is less prone to observer-dependent errors in determining lower limb alignment. Third, as mentioned earlier, the correct and reproducible measurement of the absolute tibial and femoral length is essential to estimate the growth potential. Therefore, the measurement error of the absolute femoral and tibial length needs to be elaborated.

## Methods

### Patients

**Fig. 1:**
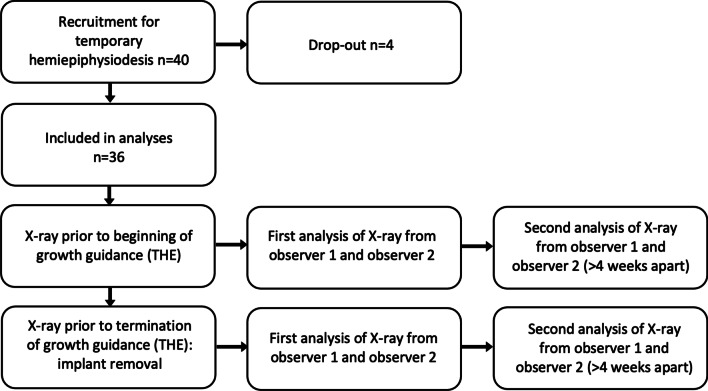
Diagram of the prospective study design. THE—temporary hemiepiphysiodesis

Patients older than 8 years of age who showed an intermalleolar distance of more than 10 cm received an anterior–posterior-full length X-ray of both legs in the standing position for objective assessment of the leg axis [7]. From the 40 patients recruited, four patients were excluded from this study due to unusable radiographs. This homogenous cohort finally consists of 36 patients (median age of 12.81 years at baseline; Table 1) with an idiopathic pathological valgus alignment of at least one knee according to the MAD of the lower limb were recruited between September 2014 and October 2021 on a voluntary basis. Patients were included if the MAD deviates more than 10 mm lateral [14] and were planned to receive THE of one or both knees [3, 12, 18]. The tension band plates used in this study were the Eight-Plates (Orthofix, Lewisville, TX, USA) or Pedi-Plates (Orthopediatrics Inc., Warsaw, IN, USA). The aim of this growth guidance technique is to straighten the leg axis. After a successful operation by means of THE is an MFA of 0° (± 2°) or an MAD of 0 mm (± 6 mm). All patients were analyzed twice, directly before guided growth intervention (72 legs) and at the time of implant removal (the same 72 legs). Accordingly, patients were deliberately measured both at a time when a lower limb malalignment was present and with corrected leg alignment to test the reliability during the entire treatment period (see Fig. 1). In total 144 legs/data sets could be evaluated.

**Table 1:** Patient characteristics (n = *36*)

Time of Investigation	Age (range) in years	Height (range) in cm	Weight (range) in kg	BMI (range) in kg/m^2^
Implantation of tension band plates using THE	12.81 (10.50–15.38)	165.89 (145.20–182.90)	63.49 (31.80–89.30)	22.86 (14.95–29.29)
Removal of tension band plates using THE	13.72 (11.32–16.35)	171.83 (150.60–187.40)	71.25 (33.80–94.50)	23.88 (14.90–32.79)

Table 1 shows the anthropometric data of the collective at the time of implantation and removal of tension band plates using the temporary hemiepiphysiodesis (THE). Values are presented as median and interquartile range in parenthesis

Exclusion criteria were: rheumatoid arthritis, anterior cruciate ligament deficiency, neuromuscular disorders, achondroplasia or hypochondroplasia, sagittal plane deformities (genu pro- and recurvatum), flexion contractures in the hip or knee joint, leg length discrepancy of more than 10 mm, avascular necrosis of the femoral head or knee condyles, history of major trauma or sport injury of the lower extremity, knee surgery within 12 months prior to inclusion in this study, chronic joint infections or previously received intraarticular corticosteroid injections.

### Radiographic malalignment analysis

The standardized, scalable, digital long leg X-rays were taken in an anterior–posterior orientation. A 25.4-mm (1 inch)-diameter metal ball was placed between the legs at the level to the bones next to the joint line of the knees and used as a reference for determining the individual magnification factor. The radiographic measurements were performed with a commercially available templating program, TraumaCad® (version 2.3.4.1; Voyant Health, Petach-Tikva, Israel). Criteria for a valid full leg length anteroposterior radiographic image were [18]: patient standing in an upright, weight-bearing position, both legs parallel to each other a shoulder width apart, fully extended knees, patellae centered over the femoral condyles pointing straight forward in order to avoid rotational errors [19, 20].

**Fig. 2:**
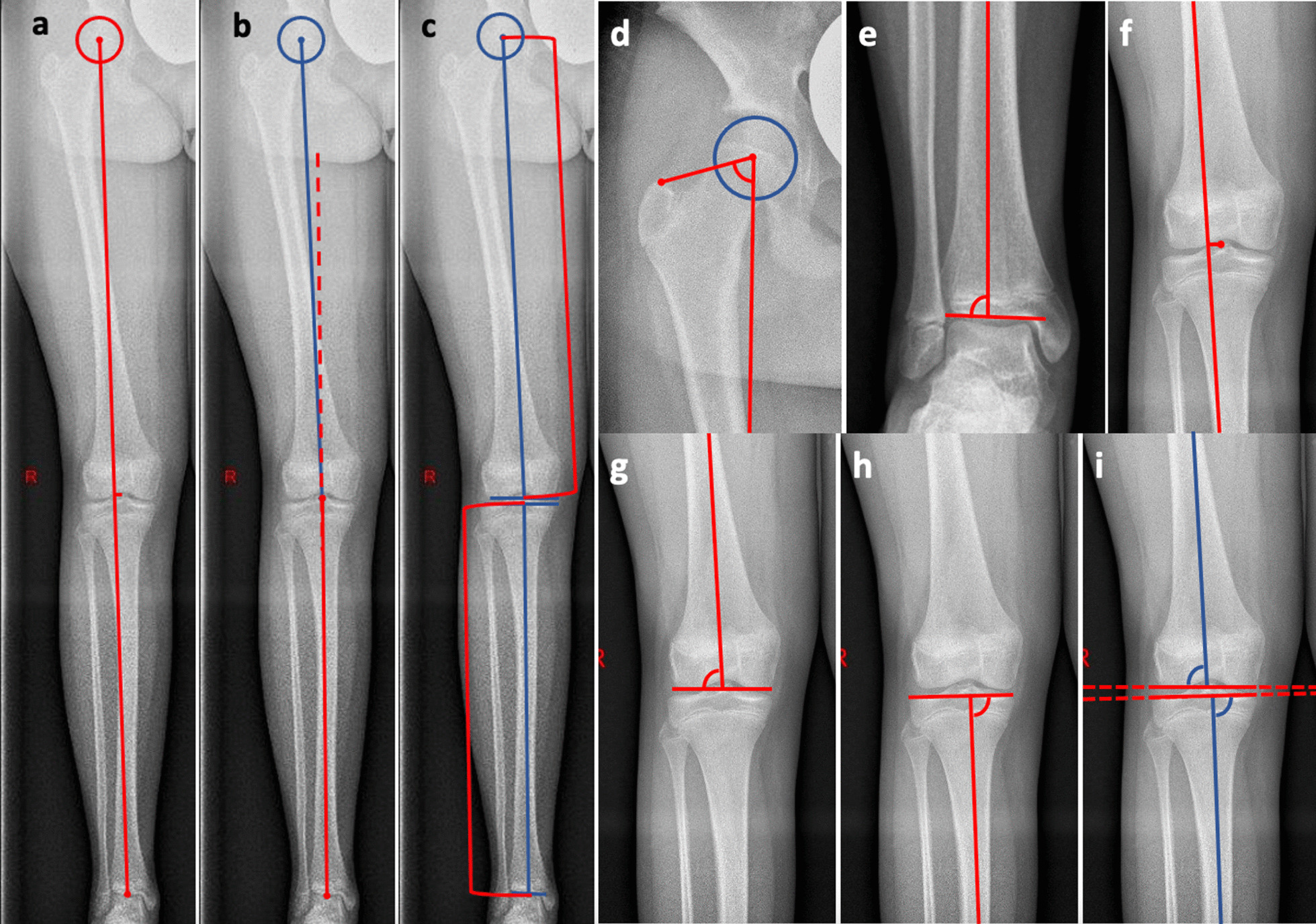
Left (Image a–c) full-length standing anterior–posterior radiograph of the right leg; a – d center of the hip (circle); **a** Mechanical axis of the limb and Mechanical Axis Deviation (MAD) (red lines); **b** Mechanical Femorotibial Angle (MFA) between mechanical femur line (blue line) and mechanical tibia line (red line); **c** length of the femur and tibia (red brackets); **d** *mLPFA* mechanical Lateral Proximal Femur Angle; **e** *mLDTA* mechanical Lateral Distal Tibia Angle; **f** detailed magnification from image a, Mechanical axis of the limb and MAD; **g** *mLDFA* mechanical Lateral Distal Femur Angle; **h** *mMPTA* mechanical Medial Proximal Tibia Angle; **i** *JLCA* Joint Line Convergence Angle [18]

Definitions of the angles measured are described below (Fig. 2):

Malalignment analysis was performed due to the principles described by Paley et. al [12, 18]: The radiographic Mechanical Femorotibial Angle (MFA) is defined as the angle formed by the line from the center of the hip to the center of the knee (mechanical femur line) and the line from the center of the knee to the center of the ankle (mechanical tibia line) (Fig. 2b) [21]. To find the center of the hip, an auxiliary circle around the femoral head was needed. The center point of the circle was determined as center of the hip. The center of the knee joint was defined as the midpoint between the center of the intercondylar region and the center of the eminentia intercondylaris. The center of the ankle was determined as the midpoint of the talar dome. Neutral alignment was defined as 0°, varus malalignment as positive angles and valgus malalignment as negative angles.

The Mechanical Axis Deviation (MAD) is defined as the distance between the center of the knee and the mechanical axis of the limb (Fig. 2a–f). Medial deviation (varus) is depicted as positive and lateral deviation (valgus) as negative values.

The mechanical Lateral Distal Femoral Angle (mLDFA) shows the angle between the mechanical femoral axis and the tangent through the most convex points of each femoral condyle (Fig. 2g).

The mechanical Medial Proximal Tibial Angle (mMPTA) depicts the angle between the mechanical tibial axis and the line along the subchondral bone from the tibial plateau (Fig. 2h).

The mechanical Lateral Proximal Femoral Angle (mLPFA) shows the angle between the mechanical femoral axis and the line from the tip of the greater trochanter to the center of the hip (Fig. 2d).

The mechanical Lateral Distal Tibial Angle (mLDTA) is defined as the angle between the mechanical axis of the tibia and the line through the medial and lateral talus shoulder (Fig. 2e).

The Joint Line Convergence Angle (JLCA) depicts the angle between the tangent of the most convex points of each femoral condyle and the line along the subchondral bone from the tibial plateau (Fig. 2i).

The length of the femur describes the distance between the center of the femoral head and the center of a line connecting the two most distal points of the medial and lateral femoral condyle (Fig. 2c) [22].

The length of the tibia describes the distance between the center of a line connecting the two most proximal points of the medial and lateral proximal tibial plateau and the center of a line.

To assess intra- and interobserver reliability, radiographs for each patient were blinded and templated twice by two independent and experienced observers (SB, MB) familiar with the templating software. The two observers are orthopedic residents with special focus on pediatric orthopedics and more than five years of experience analyzing lower limb alignments. To avoid recollection bias, the second evaluations from the same set of measurements were repeated at least 4 weeks apart, blinded to the measurements provided previously.

### Statistical analysis

To evaluate the reliability of the measurements, the intra- and interobserver intraclass correlation coefficient (ICC_2,1_) was calculated. An important advantage of ICC_2,1_ is that the coefficient can detect bias by the order in which pairs of data are compared and is therefore preferable to other correlation coefficients [23, 24]. For interobserver ICC_2,1_ the results of the first measurement of observer one are compared with the results of the first measurement of observer two. One-way random tests with absolute agreement were used for each observer to estimate intraobserver ICCs and two-way random tests with absolute agreement per set of measurements for each parameter were used to determine interobserver ICCs. ICC_2,1_ values < 0.70 indicate poor, 0.70 to 0.79 fair, 0.80 to 0.89 good, and 0.90 to 1.00 excellent reliability [23, 24].

The Shapiro–Wilk test was used to test normal distribution of the analyzed parameters. Differences between observers were tested with Wilcoxon test (nonparametric dependent variables) or paired *t*-test (parametric dependent variables). To assess the observer-dependent errors, we calculated the Standard Error of Measurements (SEM_95%_) from the interobserver ICC_2,1_.

$${\text{SEM}} = {\text{SD }} \times \sqrt {1 - {\text{ICC}}_{2,1} }$$

The SEM estimates the distribution of repeated measurements with the same instrument around their "true" value. It quantifies the precision of individual scores on tests and provides an absolute index of reliability, in contrast to ICC, which is a relative measure of reliability [25]. The SEM is directly related to the reliability of a test, i.e., the greater the SEM, the lower the reliability of the test. The range of the SEM_95%_ (e.g., MAD SEM_95%_ =  ± 3.31 mm → SEM_95%Range_ = 6.62 mm) was divided by the range of the respective norm values considered as acceptable (e.g., MAD_AcptRange_ 15 mm to 1 mm = 14 mm). This proportional error shows for which parameter, the SEM_95%_ is proportionately lowest or highest.

Statistical data analysis was performed with SPSS (version 26, IBM Corporation, New York, NY, USA). The significance level for all statistical tests was set at p ≤ 0.05. The following data were presented as mean and standard deviation (parametric variables) or median ± 25–75 percentiles (nonparametric variables).

The level of evidence of this study is a Level I, Diagnostic Study.

## Results

From September 2014 to October 2021, full-length standing anteroposterior radiographs of the legs at two different time points (implantation of tension band plates and removal of tension band plates) from 72 legs of 36 children and adolescents (16 girls and 20 boys) were included in this study. All the children and adolescents had a valgus malalignment on both sides. Table 1 shows the anthropometric data such as age, height, weight, and BMI of the collective at the time of implantation and at the time of removal of the tension band plates.

### Does the analysis of a full-length standing anteroposterior radiograph of the lower limb show a good intra- and interobserver reliability concerning lower limb alignment measurements?

The values MFA, MAD, mLPFA, mLDFA, mMPTA, mLDTA, JLCA, length of femur, and length of tibia were measured twice on 144 legs by each observer (Table 2). For both observers, the intraobserver ICC_2,1_ (Table 3) in alignment analysis was good to excellent and likewise high, ranging from 0.849 to 0.999. In contrast, fair results were only found for intraobserver ICC_2,1_ in analysis of JLCA (0.607–0.676).

**Table 2:** Lower limb measurement parameters (N = 144)

Measures	Observer one		Observer two		*p* Value
	Mean ± SD (range)	Median (25 – 75 percentiles)	Mean ± SD (range)	Median (25 – 75 percentiles)	
MFA (°)		− 2 (− 5 to 0)		− 3 (− 5 to − 1)	*p* < 0.001
MAD (mm)	− 8.69 ± 11.17 (− 38 to 22)		-9.01 ± 10.60 (− 37 to 22)		*p* = 0.013
mLPFA (°)		88 (85–91)		88 (85–92)	*p* = 0.173
mLDFA (°)		86 (84–89)		86 (84–89)	*p* < 0.001
mMPTA (°)		90 (89–92)		90 (89–91)	*p* < 0.001
mLDTA (°)		87 (84–89)		87 (85–89)	*p* < 0.001
JLCA (°)		2 (1–2)		1 (1–2)	*p* < 0.001
length of femur (mm)	474.24 ± 36.58 (387–572)		474.20 ± 36.28 (390–570)		*p* = 0.067
length of tibia (mm)		379.5 (359–402)		378 (358–401)	*p* < 0.001

Table 2 shows the mean measures from both analyses of both observers (SD = standard deviation) and the median with 25–75 percentiles

*MFA* Mechanical Femorotibial Angle; *MAD* Mechanical Axis Deviation; negative values of MFA and MAD show valgus malalignment; *mLPFA* mechanical Lateral Proximal Femur Angle; *mLDFA* mechanical Lateral Distal Femur Angle; *mMPTA* mechanical Medial Proximal Tibia Angle; *mLDTA* mechanical Lateral Distal Tibia Angle; *JLCA* Joint Line Convergence Angle, length of the femur and tibia. Parametric values: MAD and femur length; nonparametric values: MFA, mLPFA, mLDFA, mMPTA, mLDTA, JLCA and tibia length.

**Table 3:** Intraobserver reliability: Intraclass correlation coefficient ICC_2,1_ (95% confidence interval in parenthesis)

Measures	Observer one	Observer two
	Intraobserver-ICC_2,1_	Intraobserver-ICC_2,1_
MFA (°)	0.974 (0.963–0.981)	0.982 (0.972–0.989)
MAD (mm)	0.994 (0.992–0.996)	0.995 (0.993–0.996)
mLPFA (°)	0.977 (0.967–0.984)	0.961 (0.946–0.972)
mLDFA (°)	0.972 (0.962–0.980)	0.981 (0.974–0.987)
mMPTA (°)	0.889 (0.845–0.920)	0.949 (0.929–0.963)
mLDTA (°)	0.849 (0.796–0.889)	0.902 (0.866–0.929)
JLCA (°)	0.607 (0.492–0.701)	0.676 (0.612–0.757)
Femur length (mm)	0.997 (0.996–0.998)	0.999 (0.999–0.999)
Tibia length (mm)	0.997 (0.996–0.998)	0.996 (0.994–0.997)

Table 3 shows the intraobserver-ICC_2,1_ from both observers

*MFA* Mechanical Femorotibial Angle, *MAD* Mechanical Axis Deviation, *mLPFA* mechanical Lateral Proximal Femur Angle, *mLDFA* mechanical Lateral Distal Femur Angle, *mMPTA* mechanical Medial Proximal Tibia Angle, *mLDTA* mechanical Lateral Distal Tibia Angle, *JLCA* Joint Line Convergence Angle, length of the femur and tibia.

Interobserver ICC_2,1_ (Table 4) for alignment parameters (MFA, MAD, mLPFA, mLDFA, mMPTA, mLDTA, femur length and tibia length) ranged from 0.864 to 0.996. Similar to intraobserver findings, interobserver ICC_2,1_ analysis of JLCA was slightly out of line and showed fair results (0.488). It is also shown that the intraobserver ICC_2,1_ and interobserver ICC_2,1_ are slightly lower for the joint surface angles at the tibia than for the joint surface angles at the femur.

**Table 4:** Standard values and the accepted range of each lower limb parameter [12, 18], interobserver-ICC_2,1_, Standard Error of Measurement (SEM) and proportional error

Measures	Standard value	Accepted range	Interobserver-ICC_2,1_	SEM_95%_	SEM_95%Range_	Proportional error in %
MFA (°)	1.3 ± 3	6	0.949 (0.859–0.975)	± 1.39	2.78	46.33
MAD (mm)	8 ± 7	14	0.976 (0.966–0.983)	± 3.31	6.62	47.29
mLPFA (°)	90 ± 5	10	0.974 (0.964–0.981)	± 1.69	3.38	33.80
mLDFA (°)	88 ± 2.5	5	0.969 (0.954–0.979)	± 1.06	2.12	42.40
mMPTA (°)	87 ± 2.5	5	0.864 (0.816–0.901)	± 1.29	2.58	51.60
mLDTA (°)	89 ± 3	6	0.876 (0.831–0.909)	± 2.17	4.34	72.33
JLCA (°)	1 ± 1	2	0.488 (0.350–0.605)	± 1.38	2.76	138.00
Femur length (mm)	N/A	N/A	0.996 (0.994–0.997)	± 4.53	N/A	N/A
Tibia length (mm)	N/A	N/A	0.994 (0.990–0.998)	± 3.12	N/A	N/A

Interobserver-ICC_2,1_ (95% confidence interval in parenthesis) with Standard Error of Measurement (SEM) with a 95% confidence interval. For interobserver-ICC_2,1_ first measurement results of observer 1 were compared with first measurement results of observer 2

*MFA* Mechanical Femorotibial Angle; *MAD* Mechanical Axis Deviation; *mLPFA* mechanical Lateral Proximal Femur Angle; *mLDFA* mechanical Lateral Distal Femur Angle; *mMPTA* mechanical Medial Proximal Tibia Angle; *mLDTA* mechanical Lateral Distal Tibia Angle; *JLCA* Joint Line Convergence Angle, length of the femur and tibia

For values of femur length and tibia length there are no standard values and therefore no accepted ranges, SEM_95%Range_ or proportional errors are not applicable (N/A)

### Which parameter is most susceptible to observer-dependent errors in determining lower limb alignment?

As shown in Table 2, the mean MFA was −2.40° (SD ± 3.13°) for observer one and −2.81° (SD ± 3.12°) for observer two. The MAD values averaged -8.69 mm (SD ± 11.17 mm) for observer one and −9.01 mm (SD ± 10.60 mm) for observer two. The other mean values for observer one and observer two are shown in Table 2.

The mLDFA, mMPTA, mLDTA, JLCA, MFA, MAD and tibia length measurements of observer one and observer two were significant different (*p* < 0.05). No significant difference between both observers was found for femur length (*p* = 0.067) and mLPFA (*p* = 0.173).

To assess the observer-dependent errors, the SEM_95%_ was calculated (Table 4). The SEM_95%_ of both observers was found to be ± 1.39° for MFA, ± 3.31 mm for MAD, ± 1.69° for mLPFA, ± 1.06° for mLDFA, ± 1.29° for mMPTA, ± 2.17° for mLDTA and ± 1.38° for JLCA with a 95% confidence interval. In order to determine for which parameter the SEM_95%_ is proportionately lowest/highest, we divided the range of the respective SEM_95%_ (SEM_95%Range_) by the range of the different values considered acceptable (accepted range). The results (proportional error) are shown in Table 4. The proportional error of MAD was slightly higher, but comparable to the proportional error of MFA (47.29% vs. 46.33%). Looking at the relevant joint surface angles at the knee joint, we noticed that the proportional errors for the femur (mLPFA 33.80% and mLDFA 42.40%) are lower than for the tibia (mMPTA 51.60% and mLDTA 72.33%). JLCA shows the highest proportional error of 138%. Consistent to the results from the intraobserver- and interobserver ICC_2,1,_ the SEMs and the proportional errors are also larger for the articular surface angles at the tibia than at the femur.

### What is the SEM95% of the determination of the absolute femoral and tibial length, which are needed for the estimation of the existing correction potential for each bone?

The mean femur length was 474.24 mm (SD ± 36.58 mm) for observer one and 474.20 mm (SD ± 36.28 mm) for observer two. The mean tibia length was 379.80 mm (SD ± 29.85 mm) for observer one and 378.38 mm (SD ± 29.67 mm) for observer two. The statistical analysis did detect significant differences between the measurements of those two observers for the tibia length (*p* < 0.001), but not for the femur length (*p* = 0.067) (Table 2). The SEM_95%_ for the absolute values of the femoral and tibial length were ± 4.53 mm (femur) and ± 3.12 mm (tibia) (Table 4). If the SEM_95%_ of the respective bone is related to its absolute length, it can be shown that the SEM_95%_ of ± 4.53 mm for the femur is about 1.92% of the length of the femur, and ± 3.12 mm for the tibia is about 1.64% of the length of the tibia in this collective.

## Discussion

The digital measurement of biometric parameters as well as the preoperative planning of orthopedic interventions was shown to have good to excellent reliability, e.g., for Cobb angle in scoliosis [26, 27], planning of hip prostheses [28, 29] or leg axis determinations in long-leg X-rays [30–33]. The correct and reliable analysis of static parameters of the lower extremity is essential for an adequate treatment of pediatric orthopedic diseases. In addition to the clinical appearance, these measurements play a significant role in determining the indication for guided growth intervention of the lower limb using THE. In the present study the intra- and interobserver reliability for almost all lower limb alignment measurements in full-length standing anteroposterior radiographs of the frontal plane were found to be good to excellent.

Our study is the first that investigates all relevant malalignment parameters in the frontal plane in children and adolescents regarding intra- and interobserver reliability, SEM_95%_ and proportional errors in order to make the best possible and the least error-prone decision on the indication and timing of THE, on postoperative follow-up and on the right time for removal of the tension band plates after completion of growth guidance.

According to the study of Specogna et al. [34], reliability of repeated lower limb frontal plane alignment measures is high for planning a high tibial osteotomy with OA affecting the medial compartment of the knee due to varus malalignment (MFA Intra-ICC_2,1_ = 0.98; 95% CI = 0.97 − 0.99, Inter-ICC_2,1_ = 0.98; 95% CI = 0.91–0.99; MAD Intra-ICC_2,1_ = 0.98; 95% CI = 0.97 − 0.99, Inter-ICC_2,1_ = 0.97; 95% CI = 0.90–0.99). Specogna et al. [34] elucidated that the estimates of error for the measurements of MFA need to be considered with ± 1.50° and for the MAD ± 4.3 mm. These findings are largely consistent with the results of our investigation. In contrast to our findings, the mentioned study investigated MFA and MAD on radiographs collected from adult patients (mean age 44 (21–65) years). When analyzing leg axis on skeletally immature patients, Gordon et al. [31] were able to show that intra- and interobserver reliabilities for each of the measurements (mLDFA, mMPTA and MAD) were ≥ 0.90 (0.90–0.99) in children (mean age 11.2 (7.1–14.7) years) with neutral alignment and both varus or valgus malalignment regardless of the level of observer experience. Gordon et al. [31] pointed out that the overall mean interobserver differences were ± 1.4° for the mLDTA, ± 1.6° for the mMPTA and ± 3.1 mm for the MAD measurement. In a similar study, Schmale et al. [32] have shown that the intraobserver ICC values ranged from 0.27 to 0.94 for the mLDFA and from 0.88 to 0.97 for the mMPTA in patients with open distal femur and proximal tibia physes at the time of a transphyseal anterior cruciate ligament reconstruction. Furthermore, the reliability (interobserver ICC: MAD 0.92, mLDFA 0.86 and mMPTA 0.98) of leg axis measurements were rated as good to excellent. Nowicki et al. [33] evaluated the MAD, mLDFA, mMPTA and JLCA of pediatric lower extremities (mean age 13.5 years) with neutral, varus or valgus (mal)alignment but not the MFA and they did not calculate the SEM_95%_ for all relevant parameters. They could also show comparable values for the intraobserver ICC of the four measurement parameters: 0.756–0.990 for mLDFA, 0.489–0.958 for mMPTA, 0.831–0.927 for JLCA and 0.974–0.993 for MAD. Interobserver ICC values of 0.732–0.977 could be presented for the respective parameters. Feldman et al. [35] came to a similar conclusion that planning guiding treatment on those digital radiograph measurements is reliable and reproducible with intraobserver ICC of 0.77 (0.65–0.86) for mMPTA and mLDTA 0.80 (0.77–0.84). In this study, however, only the relevant joint surface angles of the tibia were comparable to our finding, because they chose to measure and analyze the anatomical angles instead of the mechanical angles. Furthermore, MAD and MFA were not investigated as well. Table 5 shows a summary of all the studies just mentioned. This is to illustrate the investigated parameters and the respective differences in our manuscript.

**Table 5:** Summary of the results of the leg axis and joint angle analyses of different studies investigating intra- and interobserver reliability and Standard Error of Measurements of lower limb radiographs

Parameters	Specogna et al.[34]	Gordon et al.[31]	Schmale et al.[32]	Nowicki et al.[33]	Feldman et al.[35]
	N = 42	N = 56	N = 15	N = 32	N = 60
*MFA*					
Intra ICC	0.98 (0.97–0.99)	N/A	N/A	N/A	N/A
Inter ICC	0.98 (0.91–0.99)	N/A	N/A	N/A	N/A
SEM	1.50	N/A	N/A	N/A	N/A
*MAD*					
Intra ICC	0.98 (0.97–0.99)	0.99 (0.99–0.99)	0.93–0.99 (0.84–1.00)	0.974–0.993 (0.946–0.996)	N/A
Inter ICC	0.97 (0.90–0.99)	0.99 (0.99–0.99)	0.92 (0.81–0.97)	0.977 (0.964–0.987)	N/A
SEM	4.3	3.1	N/A	N/A	N/A
*mMPTA*					
Intra ICC	N/A	0.99 (0.99–0.99)	0.88–0.97 (0.73–0.99)	0.489–0.958 (0.183–0.979)	0.78 (0.65–0.86)
Inter ICC	N/A	0.98 (0.97–0.98)	0.98 (0.94–0.97)	0.778 (0.670–0.869)	0.77
SEM	N/A	1.6	N/A	N/A	N/A
*mLDFA*					
Intra ICC	N/A	0.96 (0.95–0.97)	0.27–0.94 (-0.16–0.97)	0.756–0.990 (0.545–0.995)	0.80 (0.77–0.84)
Inter ICC	N/A	0.91 (0.90–0.92)	0.86 (0.68–0.95)	0.732 (0.423–0.877)	0.70
SEM	N/A	1.4	N/A	N/A	N/A
*mLPFA*					
Intra ICC	N/A	N/A	N/A	N/A	N/A
Inter ICC	N/A	N/A	N/A	N/A	N/A
SEM	N/A	N/A	N/A	N/A	N/A
*mLDFA*					
Intra ICC	N/A	N/A	N/A	N/A	N/A
Inter ICC	N/A	N/A	N/A	N/A	N/A
SEM	N/A	N/A	N/A	N/A	N/A
*JLCA*					
Intra ICC	N/A	N/A	N/A	0.831–0.927 (0.659–0.964)	N/A
Inter ICC	N/A	N/A	N/A	0.812 (0.678–0.899)	N/A
SEM	N/A	N/A	N/A	N/A	N/A

*Intra ICC* Intraobserver reliability using the intraclass correlation coefficient; *Inter ICC* Interobserver reliability using the intraclass correlation coefficient; *SEM* Standard Error of Measurement; *MFA* Mechanical Femorotibial Angle; *MAD* Mechanical Axis Deviation; *mLPFA* mechanical Lateral Proximal Femur Angle; *mLDFA* mechanical Lateral Distal Femur Angle; *mMPTA* mechanical Medial Proximal Tibia Angle; *mLDTA* mechanical Lateral Distal Tibia Angle; *JLCA* Joint Line Convergence Angle

When looking at the results of the present study, it becomes obvious that nearly all ICCs represent good to excellent correlations. Only the intra- and interobserver ICCs for JLCA are out of line and provide solely poor correlations. At a closer look at the standard values of the JLCA (1 ± 1°), it becomes apparent that a planning discrepancy of 1.38° in two successive examinations of two different observers of the same X-ray results in a relatively large discrepancy (proportional error = 138%; Table 4). This might explain why even small measurement inaccuracies become noticeable in a relatively smaller ICC. In accordance with objective 1, it can be concluded that the reliability of the individual parameters is good, except of the JLCA.

Furthermore, it was investigated which parameter is most susceptible to observer-dependent errors in determining lower limb alignment in the frontal plane. To date, there are no clear criteria for the indication of THE. The growth of the child and the development of the leg axis in children and adolescents show a high physiological range in younger patients and a typical course. While children under 2 years of age show a physiological varus alignment, a valgus leg axis develops until about 7 years of age. With advancing skeletal maturation (about 8 to 10 years of age), the range decreases significantly, which is also reflected in a decrease in the spontaneous correction of the existing leg axis deformities [36]. The results of this study refer to children aged 10 years and older, in whom the spontaneous correction potential is already reduced. Radtke et al. [8] indicate a THE of the knee at a MAD of > 10 mm, regardless of whether the deviation is medial or lateral. They were not using the MFA for indication. The same applies to other studies. Gupta et al. [37] considered a MAD of 3 mm both medial and lateral as a normal value and described a deviation of the MFA lateral to the lateral intercondyloid tubercle as valgus malalignment and a deviation medial to the medial intercondyloid tubercle as varus malalignment. In contrast, Stevens et al. [38] divide the knee joint in an anteroposterior radiograph into three zones lateral and three zones medial, mirrored at the knee joint center. Here, neither the MFA nor the MAD is directly considered. They see an indication for a THE in the case of a deviation of the mechanical bearing line in zones 2 and 3 [38].

Regarding the results of the present study, we can postulate that the SEM_95%_ of MFA, MAD, mLPFA, mLDFA, mMPTA and mLDTA are likewise high. Thus, the results suggest that if a MFA measurement of 3° valgus is assumed, an SEM_95%_ of ± 1.39° means that the actual value will range between 1.61° (non-pathologic) and 4.39° (pathologic) valgus. In addition, a MAD measurement of −10 mm lateral to the center of the knee with a SEM_95%_ of ± 3.31 mm means that the actual value ranges between −6.69 mm (non-pathologic) and -13.31 mm (pathologic) valgus. This susceptibility to error must be taken into account not only for leg alignment parameters but also for joint surface angles when interpreting malalignment analysis and must be considered when planning a surgical intervention. An inaccurate and wrong interpreted malalignment analysis may result in an incorrect diagnosis, which in turn leads to an unnecessary indication for surgical therapy. Furthermore, it could lead to termination of the guided growth intervention by a removal of the tension band plates at the wrong point of time. Considering the SEM_95%_ of the MAD and the MFA, a wrong interpretation could also be an explanation for the high rebound rates of up to nearly 50% [39]. Therefore, it must be discussed whether an overcorrection of the leg axis with a minimum of 1.39° MFA and 3.31 mm MAD should be aimed to prevent the common phenomenon of rebound by taking the respective SEM_95%_ into account. This slight overcorrection is already recommended by several authors [40, 41]. Thus, a precise malalignment measurement and the knowledge about the presented SEM_95%_ of the respective parameters is crucial for a correct surgical or nonsurgical treatment. In addition, we were able to show that by measuring the MAD and MFA, the SEM_95%_ are comparable (proportional error = 47.29% vs. 46.33%, respectively). Hereby, we consider the MAD and MFA to be equally (im)precise parameters for the determination of an axial malalignment and initiation of a growth-guiding therapy. In order to decide on which bones to perform growth guidance, mLDFA and mMPTA show distinct observer-dependent differences. The proportional error for mLDFA is lower at 42.2% than for mMPTA at 51.60%.

In addition to the observer-dependent errors, the radiological acquisition technique also influences the result of the respective angles [13, 42, 43]. When taking the full-leg radiograph, the patellae of the extended legs have to be aligned frontally (not the feet) so that the femoral condyles are positioned parallel to the frontal plane. Rotation of the leg at the time the image is taken should be avoided, as should flexion of the knee joint. In the case of increased internal rotation of the knee joints, the leg axis appears more valgus; in the case of external rotation, it appears correspondingly more varus [13]. In our study cohort, an internal quality assessment was performed first by the executing radiology assistants and subsequently by the two analyzing observers. All included patients showed qualitatively unobjectionable radiographs.

Furthermore, SEM_95%_ of tibial and femoral length was determined. Those values are essential for a proper estimation of residual growth and correction potential using the multiplier method according to Paley [15, 16, 44]. The multiplier method need an exact determination of the bone length (either tibia or femur) to give a precise information about the correction potential. We could show that the ICCs of the length determination were good and the SEM_95%_ were relatively high with ± 4.53 mm for the femur and ± 3.12 mm for the tibia. Since the femur length shows no significant difference in the measurement of two different examiners, the measurement method for determining the center of the femoral head with the aid of a circle around the femoral head with the determination of the most distal points of the two femoral condyles does not seem to be examiner-dependent and could be seen as precise when analyzing the residual growth and correction potential with the multiplier method. Due to the slightly lower SEM_95%_ in determining the tibial length, which were 1.64% of the total tibia length and ± 1.96% of the total femur length, the tibial length can be considered as precise as well. Again, it is important to know the error and include it in the analysis in predicting growth potential for each bone.

One limitation of this study is that it did not aim to identify differences in the experience of the two examiners. However, Vaishya et al. [45] showed that more experienced examiners tend to perform more precise measurements. Other studies (e.g., Gordon et al. [31]) did not find any experience-dependent differences in intra- or interobserver reliability with excellent agreement for all observers. In accordance with Vaishya et al. [45], we consider the measured values of this study to be highly reliable and reproducible and may not be improved with further experience of the observers.

In conclusion, a precise malalignment measurement and the knowledge about the presented SEM_95%_ of the respective parameters are crucial for a correct surgical or nonsurgical treatment. The susceptibility to error must be included when interpreting malalignment analysis and must be considered when planning a surgical intervention. The ICC and SEM_95%_ values in this study indicate how reproducible, reliable and precise radiological assessment of variables are obtained from an X-ray. In general, with any individual measurement, it should be understood that a measurement can never be determined exactly but is always subject to a certain measurement error or measurement inaccuracy. The same applies to anatomical standard values, which also have their physiological range. The results of this study elucidate that MAD and MFA show no different susceptibility to observer-dependent errors. In addition, due to no significant difference in determining the femoral length between two different observers and a lower SEM_95%_ for tibial length by two different observers, both femoral and tibial length may be considered precise when analyzing the residual growth and correction potential for the respective bone, for instance, with the multiplier method. This study shows good to excellent intra- and interobserver ICCs for all leg alignment parameters and joint surface angles, except for JLCA. Furthermore, according to the SEM_95%_ and proportional errors the determination of joint surface angels of the femur seems to be more precise than for the tibia. In unclear or borderline cases with marginal MAD and MFA, instrumented gait analysis and the determination of dynamic joint loading [4, 5, 11] should be included to determine whether surgical or nonsurgical treatment should be preferred.

## Data Availability

The datasets used and/or analyzed during the current study are available from the corresponding author on reasonable request.

## Abbreviations

OA: Osteoarthritis
THE: Temporary hemiepiphysiodesis
SEM: Standard Error of Measurement
MAD: Mechanical Axis Deviation
MFA: Mechanical Femorotibial Angle
JLCA: Joint Line Convergence Angle
mLPFA: Mechanical Lateral Proximal Femur Angle
mLDFA: Mechanical Lateral Distal Femur Angle
mMPTA: Mechanical Medial Proximal Tibia Angle
mLDTA: Mechanical Lateral Distal Tibia Angle
ICC: Intraclass correlation coefficient
SD: Standard deviation
